# The Novel Role of MIF in the Secretion of IL-25, IL-31, and IL-33 from PBMC of Patients with Rheumatoid Arthritis

**DOI:** 10.3390/molecules26164968

**Published:** 2021-08-17

**Authors:** Samuel García-Arellano, Luis Alexis Hernández-Palma, Sergio Cerpa-Cruz, Gabriela Athziri Sánchez-Zuno, Melva Guadalupe Herrera-Godina, José Francisco Muñoz-Valle

**Affiliations:** 1 Instituto de Investigación en Ciencias Biomédicas (IICB), CUCS, Universidad de Guadalajara, Guadalajara 44340, Jalisco, Mexico; samuel.garcia4566@academicos.udg.mx (S.G.-A.); luis.hernandez4360@academicos.udg.mx (L.A.H.-P.); athziri.sanchez@alumnos.udg.mx (G.A.S.-Z.); melva.herrera@academicos.udg.mx (M.G.H.-G.); 2 Departamento de Reumatología, Hospital Civil de Guadalajara “Fray Antonio Alcalde”, Guadalajara 44280, Jalisco, Mexico; sacer04@prodigy.net.mx

**Keywords:** MIF, IL-25, IL-31, IL-33, PBMC, rheumatoid arthritis

## Abstract

Rheumatoid arthritis (RA) is an autoimmune inflammatory joint disease with complex pathogenesis associated with cytokine dysregulation. Macrophage migration inhibitory factor (MIF) plays a role in systemic inflammation and joint destruction in RA and could be associated with the secretion of other immune-modulatory cytokines such as IL-25, IL-31, and IL-33. For the above, our main aim was to evaluate the IL-25, IL-31, and IL-33 secretion from recombinant human MIF (rhMIF)-stimulated peripheral blood mononuclear cells (PBMC) of RA patients. The rhMIF and lipopolysaccharide (LPS) plus rhMIF stimuli promote the secretion of IL-25, IL-31, and IL-33 (*p* < 0.05) from PBMC of RA patients. The study groups, the different stimuli, and the interaction between both showed a statistically significant effect on the secretion of IL-25 (*p* < 0.05) and IL-31 (*p* < 0.01). The study of the effect of the RA patient treatments and their interaction with the effect of stimuli did not show an interaction between them. In conclusion, our study generates new evidence for the role of MIF in the secretion of IL-25, IL-31, and IL-33 and its immunomodulatory effect on RA.

## 1. Introduction

Rheumatoid arthritis (RA) is an autoimmune chronic disease associated with high levels of proinflammatory cytokines, secreted mainly by mononuclear cells such as T lymphocytes and macrophages [1]. Macrophage migration inhibitory factor (MIF) is an inflammatory mediator that participates in the regulation of the immune response and plays an important role in the joint destruction process in RA [2]. In macrophages and synovial fibroblasts, MIF induces the expression of tissue-destructive cytokines and mediators, including tumor necrosis factor-α (TNF-α), interleukin (IL)-1β, IL-6, IL-8, and some matrix metalloproteinases (MMP) such as MMP-1 and MMP-3 [3].

In the pathogenetic process of RA, a network of cytokines mediates joint damage and must therefore be evaluated as a whole. In addition to MIF, other cytokines such as IL-25, IL-31, and IL-33 are involved in the pathogenesis of different inflammatory diseases, including RA. It has been shown in a mouse model of collagen-induced arthritis that high expression of IL-25 is related to a regulatory mechanism in the presence of high levels of IL-17 [4], and it has been found that IL-33 can intensify collagen-induced arthritis in experimental models [5]. Complementary with this, high levels of IL-25 in the synovial fluid and serum of RA patients have been reported [4] and evidence for the relationship between IL-33 and RA exists, since IL-33 levels are higher in patients compared to healthy subjects [6].

Further to the above, although the biological effect of IL-31 is currently unclear and its participation in some inflammatory processes is controversial, it has been shown to stimulate the secretion of proinflammatory cytokines, such as IL-6, and different chemokines and MMP, mainly in skin disorders [7,8], suggesting that this cytokine has a proinflammatory function and could therefore play a role in RA pathogenesis.

Additionally, MIF significantly upregulates the expression of proinflammatory molecules by activating extracellular signal regulated kinase (ERK), p38, and c-Jun N-terminal kinase (JNK) pathways [3], which are named after three molecules that belong to the mitogen-activated protein kinase (MAPK) family [9]. Some studies have reported that the MAPK pathway is associated with IL-25 [10], IL-31 [11], and IL-33 [11,12,13] production. Therefore, MIF could be a potential inducer of these cytokines.

Based on the foregoing, the main purpose of this study was to evaluate whether recombinant human MIF (rhMIF) promotes the secretion of IL-25, IL-31, and IL-33 in peripheral blood mononuclear cells (PBMC) from control subjects (CS) and RA patients and to recognize the possible relationship between these four cytokines and their role in RA. Our results indicate that MIF participates in the secretion of IL-25, IL-31, and IL-33 in RA as well as an interaction between the type of stimulus and the secretion of these cytokines.

## 2. Results

### 2.1. Demographic and Clinical Characteristics of Donors of PBMC

Eighteen CS and eighteen RA patients were included in this study. All participants were female, and the range of age in both groups was 25 to 60 years (no significant differences were found between groups). In the RA group, the mean disease duration was 6.6 + 3 years, and all patients had high disease activity according to the DAS-28 index. In addition, 50% of patients received chloroquine, 89% methotrexate, and 44% sulfasalazine for the treatment of their disease (Supplementary Table S1).

### 2.2. Effect of LPS and rhMIF on IL-25, IL-31, and IL-33 Secretion from PBMC of CS and RA Patients

**Figure 1:**
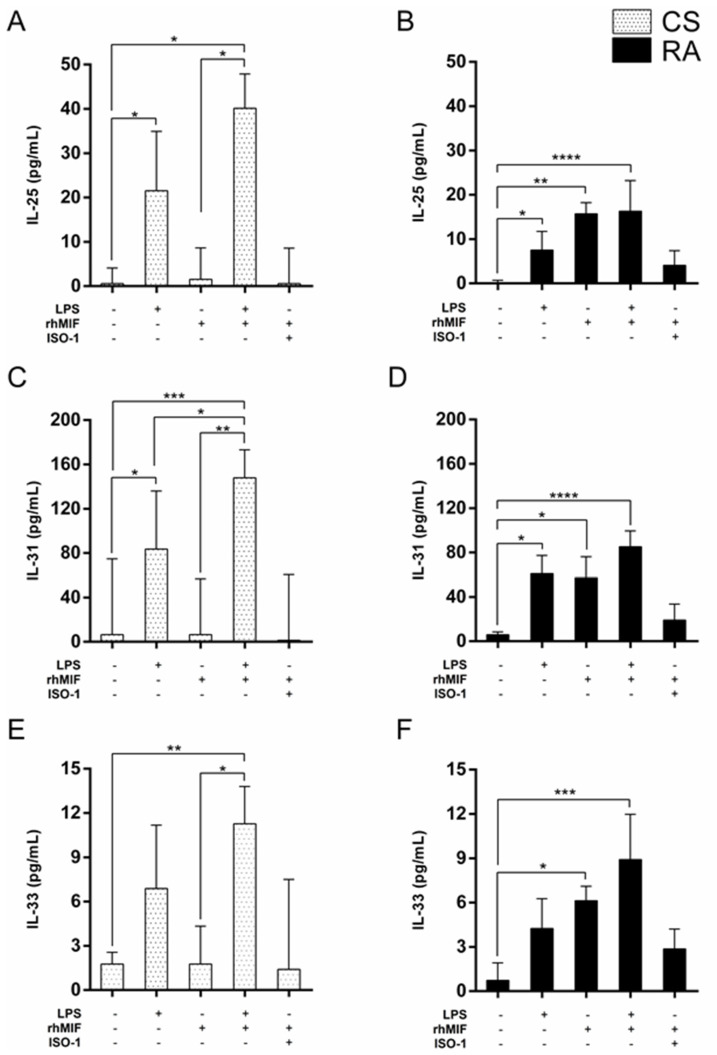
Levels of IL-25, IL-31, and IL-33 in supernatants of PBMC from CS (white with black dots) and RA patients (black) after 24 h of stimulation with LPS, rhMIF, and ISO-1 in different combinations. The soluble levels of IL-25 (**A**,**B**), IL-31 (**C**,**D**), and IL-33 (**E**,**F**) in the supernatant of unstimulated PBMC and stimulated PBMC with LPS (30 ng/mL), rhMIF (200 ng/mL), LPS (30 ng/mL) plus rhMIF (200 ng/mL), and rhMIF (200 ng/mL) plus ISO-1 (100 µM) were determined. The plus and minus signs (+ and −), represent the presence and absence of the reagent mentioned at the bottom of each graph, respectively. All assays were set up in triplicate (*n* = 18 per group). Data provided in median and interquartile range 25th–75th. Statistical analysis was performed using the Kruskal–Wallis test followed by Dunn’s multiple comparison test. In all Kruskal–Wallis tests, *p* < 0.05 was obtained. * *p* < 0.05, ** *p* < 0.01, *** *p* < 0.001, **** *p* < 0.0001.

To study the functional role of MIF on IL-25, IL-31, and IL-33 secretion, PBMC obtained from CS and RA patients were cultivated for 24 h in the absence or presence of rhMIF (unstimulated PBMC and rhMIF-stimulated PBMC, respectively). Additionally, we used lipopolysaccharide (LPS) as a positive control for PBMC stimulation to evaluate the capacity of cells to respond to the stimulus and to identify whether there is a differential response between groups. We observed that LPS significantly promoted the secretion of IL-25 and IL-31 but not IL-33 secretion from PBMC in both groups (Figure 1), whereas rhMIF only significantly increased the secretion of IL-25 [median 15.73, with interquartile range (IQR) 1.12–18.25 pg/mL, *p* < 0.01], IL-31 (median 57.12, IQR 47.43–76.16 pg/mL, *p* < 0.05), and IL-33 (median 6.13, IQR 2.07–7.11 pg/mL, *p* < 0.05) from PBMC of RA patients (Figure 1B,D,F).

Furthermore, the PBMC were stimulated with rhMIF plus ISO-I (a MIF inhibitor) to identify if the effect of rhMIF decreased. A slight increase in the secretion of IL-25, IL-31, and IL-33 from PBMC of CS and RA patients was observed, but it was not significant (Figure 1).

### 2.3. Effect of Interaction between LPS Plus rhMIF on IL-25, IL-31, and IL-33 Secretion from PBMC of CS and RA Patients

We evaluated if a synergistic interaction between LPS and rhMIF exists. Our study showed that the secretion of IL-25, IL-31, and IL-33 after exposure of PBMC from CS and RA to LPS and rhMIF was significant (Figure 1). Besides, a 2 × 2 factorial design was developed to determine the interaction between LPS and rhMIF on the secretion of IL-25, IL31, and IL-33 in PBMC obtained from both study groups. This analysis corroborated that both LPS and rhMIF alone can generate a different response between PBMC from CS and RA patients in the secretion of IL-25, IL-31, and IL-33, and that LPS in conjunction with rhMIF generates an additive effect on PBMC from both groups but no synergistic interaction (Table 1).

### 2.4. Analysis of Interactions between Groups of Study or Treatments with Stimuli Effects

**Figure 2:**
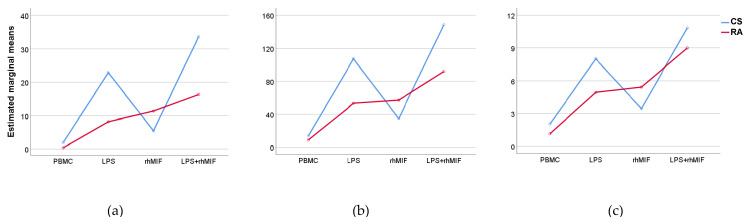
Interactions between the effect of stimuli and group of study on the secretion of the IL-25, IL-31, and IL-33 in the supernatants of PBMC from CS and RA patients after 24 h of stimulation with LPS and rhMIF: (**a**) IL-25. An effect of the type of stimulus, the study group, and their interaction on IL-25 secretion was found. Post hoc testing revealed differences in LPS (*p* < 0.01) and LPS plus rhMIF (*p* < 0.001) vs. unstimulated PBMC; (**b**) IL-31. An effect of the type of stimulus, the study group, and their interaction on IL-31 secretion was found. Post hoc testing revealed differences in LPS (*p* < 0.001), rhMIF (*p* < 0.05) and LPS plus rhMIF (*p* < 0.001) vs. unstimulated PBMC; (**c**) IL-33. Only the effect of the type of stimulus (*p* < 0.001) but no effect of the study group and no interaction between these on IL-31 secretion was observed. Post hoc testing revealed differences in LPS (*p* < 0.01) and LPS plus rhMIF (*p* < 0.001) vs. unstimulated PBMC. Data provided in estimated marginal means. Statistical analysis was performed using the two-way ANOVA followed by Tukey post hoc test.

**Figure 3:**
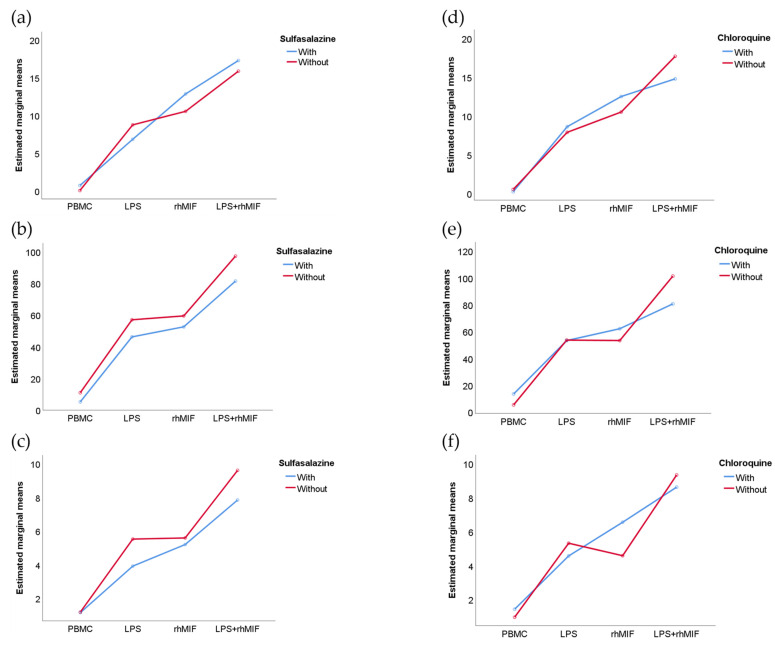
Interactions between the effect of stimuli and used of sulfasalazine (**a**–**c**) or chloroquine (**d**–**f**) treatments on the secretion of IL-25, IL-31, and IL-33 in supernatants of PBMC from RA patients after 24 h of stimulation with LPS and rhMIF. In the graphs, the blue line (With) refers to consuming the drug by RA patients and the red line (Without) refers to not consuming the drug evaluated as a factor. No effect of the type of stimulus or different treatments on the secretion of the IL-25, IL-31, and IL-33 was found. Data provided in estimated marginal means. Statistical analysis was performed using the two-way ANOVA followed by Tukey post hoc test.

The results of interactions between the study groups (factor A) and different stimuli (factor B) showed that factor A, factor B, and their interaction (A × B) have a statistically significant effect on the secretion of IL-25 (*p* < 0.05, *p* < 0.001, and *p* < 0.01, respectively) and IL-31 (*p* < 0.01, *p* < 0.001, and *p* < 0.01, respectively), but only the effect of different stimuli showed a significant change (*p* < 0.001) on the secretion of IL-33 (Figure 2). The use of different medications by patients with RA could intervene in the cellular response to stimulation with LPS or rhMIF, for which this variable was analyzed as a factor. Furthermore, it is important to mention that methotrexate, the drug most used by the patients with RA, was not considered in this analysis because the minimum data per group was not covered. We observed that in the RA patient group, the sulfasalazine and chloroquine drugs showed no interactions with different stimuli (Figure 3).

## 3. Discussion

RA is a rheumatic disease characterized by synovitis and systemic inflammation in which a complex network of different cytokines is known to be involved in tissue damage, but the exact mechanism of disease pathogenesis is still unknown [1]. The cytokine MIF has been associated with the inflammatory process in RA due to its important regulatory role in the immune response [2]. Regarding this, it has been described that MIF can be stimulated by the persistent proinflammatory response in RA, and that it can promote the activation of phospholipase A2 (PLA2) and the production of cyclooxygenase-2 (COX-2) and prostaglandin E2 (PGE2) in multiple immune cells that are associated with joint inflammation and the promotion of the production of proinflammatory cytokines that play a crucial role in early and established RA, such as TNF-α, interferon (IFN)-γ, IL-1β, IL-6 [3], and IL-17 [4].

In our research group, we have been interested in knowing the cellular response mediated by MIF in RA in such a way that we set out to investigate whether there is a relationship between MIF and the production of other cytokines recently related to the pathogenesis of this disease, such as IL-25, IL-31, and IL-33. Furthermore, the fact that LPS is a powerful activator of the immune system and that its interaction with monocytes and monocyte-derived macrophages via TLR-4 receptor results in cellular activation and also in the secretion of cytokines related to Th1, Th2, or Th17 profiles [5,6] motivated us to investigate the role it could have in the secretion of IL-25, IL-31, and IL-33.

Our results show that IL-25 levels increased in the supernatants of PBMC from CS and RA after stimulation with LPS, providing the first report on said response in CS and RA PBMC and supporting what was described by Liu et al. [7] on the effect of LPS on IL-25 production (studied in bronchial epithelial cells in vitro). The cytokine IL-25 (also named IL-17E) is a member of the IL-17 family [7] that can affect or benefit the host depending on how it interacts with other components of the immune response and the cell through which it exerts its effects [8]. This cytokine is produced by different immune cells such as T helper (Th) 2 cells, mast cells derived from bone marrow, and alveolar macrophages, among others [7,8], and can upregulate Th2-mediated immune responses and downregulate those mediated by Th1 and Th17 in diseases such as inflammatory bowel disease, multiple sclerosis, and diabetes mellitus [7]. It is also important to consider that IL-25 protects against LPS-induced lethal endotoxemia [14] in such a way that there could be a regulation between these cytokines in inflammatory processes associated with the presence of LPS.

We also observed that IL-31 levels significantly increase in LPS-stimulated PBMC in both study groups. No published data have previously described the direct effect of LPS on PBMC from RA patients in the induction of IL-31 secretion; however, other studies showed that LPS induces IL-31 secretion in astrocytes [11] and RAW264.7 cells [15]. Furthermore, it was shown that IL-31 production from several cell sources seems to be dependent on IL-4, which plays an important role in Th2 polarization from naive T cells and in the maintenance of Th2 activation [16,17]. On the other hand, several studies have reported that LPS induces IL-4 production by macrophages [18] and, thus, LPS could be indirectly inducing IL-31 production.

Surprisingly, IL-33 concentrations did not change significantly after LPS stimulation on PBMC from CS and RA patients in comparison with the unstimulated cells. The reasons for these inconsistencies are not clear, although the data may reflect the variable responsiveness of different cell types. Similarly, in a study of monocyte culture, IL-33 could not be detected in supernatants even after LPS stimulation [19]. IL-33 possesses dual roles both as a traditional extracellular cytokine and as an intracellular nuclear factor with transcriptional regulatory properties. The pathophysiological role of IL-33 as a nuclear factor is not fully understood, but it is suggested that nuclear IL-33 sequesters nuclear factor-kB (NF-kB) and reduces NF-kB-triggered gene expression to dampen proinflammatory signaling [20]. Therefore, this could indicate that the function, if any, of IL-33 in activated monocytes is primarily intracellular.

It has been reported that IL-33 plays an important immune role associated with the Th2 response, significantly stimulating the secretion of IL-5 and IL-13 by Th2 polarized cells. The IL-33 receptor is a complex formed by ST2 protein and by accessory protein for IL-1 receptor (IL-1RAcP), both expressed by many resting or activated immune cells including Th2 cells, mast cells, basophils, macrophages, dendritic cells, CD8 T cells, and B cells [21]. In addition to immune cells, ST2 is also expressed in many tissues and organs, suggesting the involvement of the IL-33/ST2 pathway in the pathogenesis of many diseases. Therefore, it is likely that IL-33 can play either a pro- or anti-inflammatory role depending on the specific disease and immune context [22,23].

Likewise, one of the most relevant findings of this study is that MIF was demonstrated to stimulate the secretion of IL-25, IL-31, and IL-33 from PBMC of RA patients but this does not occur in PBMC from CS. In this regard, it is known that MIF can act through CD74 and CD44 interaction [24], as well as CXC chemokine receptor (CXCR) 2, CXCR4, and CXCR7 [25,26]. It is possible that these receptors mediate different responses in which MIF would induce the secretion of IL-25, IL-31, and IL-33, but it is important to investigate the direct effect of these receptors on the response of PBMC to different concentrations of rhMIF. In our research group, it has been proposed that the differential response to rhMIF between CS and RA could be mediated by a higher expression of MIF receptors in the PBMC of patients, since it has been reported that RA patients have high CXCR4 expression levels in synovial tissue CD4(+) memory T cells [27] and, therefore, something similar could be happening with the other MIF receptors.

It is important to consider that MIF and LPS share signaling pathways for MAPK and JNK [3,7] and that MIF regulates the expression of the LPS receptor [3] in such a way that MIF could favor a higher LPS response in the PBMC from participants. This information could explain the additive effect observed when stimulating PBMC with LPS plus rhMIF in our study. It could also be hypothesized that increased secretion of IL-25 and IL-33 after LPS plus rhMIF stimulation is related to a regulatory mechanism to decrease the production of proinflammatory cytokines because IL-25 directly can stimulate Th2 cell differentiation through to IL-4 in a STAT6-dependent manner [28], and IL-33 can promote polarization of the anti-inflammatory M2 macrophages which, in turn, leads to the expansion of Treg cell populations that suppress the proinflammatory response [29].

Although we have proposed that the rhMIF differential response in the PBMC from RA patients may be due to a higher expression of MIF receptors, we must also consider that the response observed between the study groups and the response to LPS could be due to a lower expression of the LPS receptor because it has been reported that different treatments in RA patients (chloroquine, methotrexate, and sulfasalazine) can affect the response to LPS through decreased mRNA expression of TLR4 [30,31,32,33], and this occurs possibly by inactivation of NF-κB [30].

It is relevant to add that our study has some limitations. Among them, it is mentioned that it is crucial to include patients with mild and moderate activity who donate PBMC and, also, to quantify the different subpopulations present in the PBMC or stimulated by this condition. In addition, inhibitors of IL-4 and of other cytokines involved in the stimulation of secretion of the studied cytokines could be used in the present investigation to define whether MIF is a direct inducer of the observed response and, if so, explain through which receptors and by which signaling pathways it carries out its activity. Finally, it could also be evaluated whether there is IL-33 sequestration upon stimulation with LPS. For all the above, despite showing novel data, the reported results should be interpreted with caution and confirmed by other experiments.

In conclusion, our results contribute important new information that MIF participates in the secretion of IL-25, IL-31, and IL-33, which plays an essential role in Th2 polarization. Although we have reported that MIF favors the secretion of cytokines related to the Th1 and Th17 profile [34,35], the present study indicates that MIF could also be associated with the induction of a Th2 profile, which acts as a negative regulatory circuit, trying to counteract the inflammatory processes observed in RA. However, further studies are needed to explain how MIF induces the expression of IL-25, IL-31, and IL-33 at diverse stages of the disease.

## 4. Materials and Methods

### 4.1. Participants

In the present study, RA patients, defined by the 2010 American College of Rheumatology/European League Against Rheumatism (ACR/EULAR) criteria [36], were identified in the Rheumatology Department of the Hospital Civil de Guadalajara “Fray Antonio Alcalde,” Jalisco, México. Demographic data including age, sex, and time since diagnosis were collected. A tender joint count (0–28), swollen joint count (0–28), patient’s best global assessment, and erythrocyte sedimentation rate (ESR) were performed to calculate the Disease Activity Score 28-joints (DAS28-ESR). The use of disease-modifying anti-rheumatic drugs (DMARDs) was recorded. The CS group conformed to the general population with no previous diagnosis of RA or other chronic inflammatory diseases. Ethical approval for this study was obtained from a local Ethics Committee (Universidad de Guadalajara; CI-11908), and written informed consent was obtained from all subjects before the study. The planning conduct and reporting of human research were performed under the Declaration of Helsinki amendments. 

### 4.2. Isolation of PBMC and Culture Protocol

The PBMC were isolated from whole blood samples of CS and RA patients by Lymphoprep™ (NYCOMED; *p* 1.077 ± 0.001 g/mL) density gradient centrifugation. Mononuclear cells layer collected from the interphase were washed and resuspended in RPMI-1640 medium (Life Technologies, Grand Island, NY, USA; ATCC modification) containing antibiotic/antimycotic (50 U/mL penicillin and 50 µg/mL streptomycin, 1% (*v*/*v*); Sigma Aldrich, St. Louis, MO, USA). The cell viability was assessed with the Trypan blue exclusion method. Cell viability was never below 95%, even after 24 h of culture. Freshly isolated PBMC were plated in triplicate into cell culture 96-well plates at a density of 1 × 10^5^ cells/well with 30 ng/mL LPS (*Escherichia coli* O55:B5; CAT-L2880, Sigma-Aldrich), 200 ng/mL rhMIF (CAT-289 MF, R&D Systems™, Minneapolis, MN, USA), or LPS plus rhMIF. To decrease rhMIF response, ISO-I (CAS 478336-92-4-Calbiochem^®^ EMD Millipore, Billerica, MA, USA), an inhibitor of MIF, was added at 100 µM concentration in another assay. PBMC incubated with medium alone were used as a control. The cells were incubated for 24 h at 37 °C in 5% CO_2_. Subsequently, supernatants were collected and stored at −80 °C until further processing.

### 4.3. Cytokine Assays

The supernatants of PBMC culture were analyzed for the detection of three cytokines, IL-25, IL-31, and IL-33. Sample supernatants were assayed by multiplex immunoassay according to the manufacturer’s instructions using the Bio-Plex Pro™ Human Cytokine Screening Panel (Bio-Rad Laboratories, Inc., Hercules, CA, USA). A Bio-Plex^®^ MAGPIX system (Luminex, Austin, TX, USA) was used to read the multiplex assay. The cytokine concentrations were calculated to perform a five-parameter logistic curve. All samples were analyzed in duplicate.

### 4.4. Statistical Analysis

Data analysis was performed using statistical software SPSS v.27 (Chicago, IL, USA) and GraphPad Prism v.6.01 (La Jolla, CA, USA) software. The normality of data distribution was evaluated using the Shapiro–Wilk test. For the descriptive analysis, continuous variables with normal distribution were expressed as mean ± standard deviation (SD), and non-normal distribution was expressed as medians with IQR 25th–75th. To evaluate differences in cell culture stimuli, the Kruskal–Wallis test and Dunn’s adjustment for multiple comparisons were used. The interactions between LPS and rhMIF were studied by two-way ANOVA and the interactions between groups of study or treatments with stimuli effects by two-way ANOVA followed by Tukey post hoc test. In all cases, *p*-value < 0.05 was considered statistically significant.

## Figures and Tables

**Table 1:** ANOVA parameters for the characterization of the 2 × 2 factorial design for the effect of LPS plus rhMIF on IL-25, IL-31, and IL-33 secretion from PBMC.

Investigated Parameters	Sum of Squares (SS)	Mean of Squares (MS)	F	*p* Value	Sum of Squares (SS)	Mean of Squares (MS)	F	*p* Value
	CS				RA			
IL-25								
rhMIF	460	460	2.163	0.151	830.2	830.2	19.025	**<0.001**
LPS	5440.8	5440.8	25.578	**<0.001**	370.7	370.7	8.495	**0.006**
rhMIF + LPS	117.9	117.9	0.554	0.462	17.8	17.8	0.407	0.528
Error	6806.9	212.7			1396.4	43.637		
Total SS	21,988.9				5565.4			
IL-31								
rhMIF	8690.6	8690.6	4.2	**0.049**	16,992	16,992	32.472	**<0.001**
LPS	96,602.9	96,602.9	46.692	**<0.001**	14,176	14,176	27.091	**<0.001**
rhMIF + LPS	905.8	905.8	0.438	0.513	208.3	208.3	0.398	0.533
Error	66,206.7	2069			16,745	523.3		
Total SS	380,120				147,769			
IL-33								
rhMIF	40.6	40.6	2.672	0.112	156.2	156.2	16.427	**<0.001**
LPS	402.3	402.3	26.453	**<0.001**	122.6	122.6	12.898	**0.001**
rhMIF + LPS	4.5	4.5	0.295	0.591	0.14	0.14	0.015	0.903
Error	486.7	15.209			304.2	9.5		
Total SS	2266.1				1530.8			

A two-way ANOVA was used to assess the effect at a 5% level of significance. The degrees of freedom were one in all cases. Significant levels are highlighted in bold.

## Data Availability

The data presented in this study are not publicly available.

## Supplementary Materials

The following are available online. Table S1: Demographic and clinical characteristics of CS and RA patients.

## Sample Availability

Sample of the compounds are not available from the authors.
