# Supplementary material for: The Novel Role of MIF in the Secretion of IL-25, IL-31, and IL-33 from PBMC of Patients with Rheumatoid Arthritis
Source: Molecules. 2021 Aug 17;26(16):4968. doi: 10.3390/molecules26164968 (PMC8398282; doi:10.3390/molecules26164968)
Supplement: Supplementary file 1 [file molecules-26-04968-s001.zip › molecules-1294362-supplementary.pdf]

molecules

MDPI

Supplementary Materials

The Novel Role of MIF in the Secretion of IL-25, IL-31, and IL-33 from PBMC of Patients with Rheumatoid Arthritis

Samuel García-Arellano [1], Luis Alexis Hernández-Palma [1], Sergio Cerpa-Cruz [2], Gabriela Athziri Sánchez-Zuno [1], Melva Guadalupe Herrera-Godina [1] and José Francisco Muñoz-Valle [1,*]

1 Instituto de Investigación en Ciencias Biomédicas (IICB), CUCS, Universidad de Guadalajara, Guadalajara, 44340 Jalisco, México; samuel.garcia4566@academicos.udg.mx (S.G.-A.); luis.hernandez4360@academicos.udg.mx (L.A.H.-P.); athziri.sanchez@alumnos.udg.mx (G.A.S.-Z.); melva.herrera@academicos.udg.mx (M.G.H.-G.)

2 Departamento de Reumatología, Hospital Civil de Guadalajara "Fray Antonio Alcalde", Guadalajara, 44280 Jalisco, México; sacer04@prodigy.net.mx

* Correspondence: drjosefranciscomv@cucs.udg.mx; Tel.: +52 3336266471

Table S1. Demographic and clinical characteristics of CS and RA patients.

**Variables**	**CS (n = 18)**	**RA (n = 18)**	p
Age (years)*	47 ± 13	48 ± 10	0.45
ESR (mm/h)*	16.0 ± 10	35 ± 15	**0.03**
Disease duration (years)*	-	6.6 ± 3	-
DAS28[+]	-	5.8 (5.2–6.0)	-
Treatment°			
Chloroquine[a]	-	50 (9/18)	-
Methotrexate[b]	-	89 (16/18)	-
Sulfasalazine[c]	-	44 (8/18)	-

*Data is shown as the mean ± standard deviation (SD), [+]Data is shown as median (interquartile ranges 25th-75th), °Data shown as % (n), [a]150 mg per day, [b]10 mg per week, [c]500 mg per day. Student's t-test was used to evaluate the differences between groups. Abbreviations: CS, control subjects; DAS28, disease activity score 28; ESR, erythrocyte sedimentation rate; RA, rheumatoid arthritis.
